# Mice deficient in complement C3 are protected against recognition memory deficits and astrogliosis induced by status epilepticus

**DOI:** 10.3389/fnmol.2023.1265944

**Published:** 2023-11-14

**Authors:** Nicole D. Schartz, Alisha Aroor, Yibo Li, Nicole Pinzón-Hoyos, Amy L. Brewster

**Affiliations:** ^1^Department of Psychological Sciences, Purdue University, West Lafayette, IN, United States; ^2^Department of Geriatrics, University of Arkansas for Medical Sciences, Little Rock, AR, United States; ^3^Department of Biological Sciences, Southern Methodist University, Dallas, TX, United States

**Keywords:** complement, epilepsy, memory, astrocytes, pilocarpine, hippocampus, C3, status epilepticus

## Abstract

**Introduction:**

Status epilepticus (SE) can significantly increase the risk of temporal lobe epilepsy (TLE) and cognitive comorbidities. A potential candidate mechanism underlying memory defects in epilepsy may be the immune complement system. The complement cascade, part of the innate immune system, modulates inflammatory and phagocytosis signaling, and has been shown to contribute to learning and memory dysfunctions in neurodegenerative disorders. We previously reported that complement C3 is elevated in brain biopsies from human drug-resistant epilepsy and in experimental rodent models. We also found that SE-induced increases in hippocampal C3 levels paralleled the development of hippocampal-dependent spatial learning and memory deficits in rats. Thus, we hypothesized that SE-induced C3 activation contributes to this pathophysiology in a mouse model of SE and acquired TLE.

**Methods:**

In this study C3 knockout (KO) and wild type (WT) mice were subjected to one hour of pilocarpine-induced SE or sham conditions (control; C). Following a latent period of two weeks, recognition memory was assessed utilizing the novel object recognition (NOR) test. Western blotting was utilized to determine the protein levels of C3 in hippocampal lysates. In addition, we assessed the protein levels and distribution of the astrocyte marker glial fibrillary acidic protein (GFAP).

**Results:**

In the NOR test, control WT + C or C3 KO + C mice spent significantly more time exploring the novel object compared to the familiar object. In contrast, WT+SE mice did not show preference for either object, indicating a memory defect. This deficit was prevented in C3 KO + SE mice, which performed similarly to controls. In addition, we found that SE triggered significant increases in the protein levels of GFAP in hippocampi of WT mice but not in C3 KO mice.

**Discussion:**

These findings suggest that ablation of C3 prevents SE-induced recognition memory deficits and that a C3-astrocyte interplay may play a role. Therefore, it is possible that enhanced C3 signaling contributes to SE-associated cognitive decline during epileptogenesis and may serve as a potential therapeutic target for treating cognitive comorbidities in acquired TLE.

## 1. Introduction

Epilepsy is a neurological disorder characterized by an imbalance of excitatory-inhibitory neurotransmission in the brain that results in the generation of spontaneous recurrent seizures (Stafstrom and Carmant, 2015). It is estimated that 30−40% of individuals with epilepsy have drug-resistant seizures (Xue-Ping et al., 2019) and that > *50*% develop cognitive and psychiatric comorbidities such as learning difficulties, memory deficits, anxiety, and depression (Helmstaedter and Witt, 2017; Paudel et al., 2018). Unfortunately, efficacious seizure-reducing drugs do not help improve these comorbidities, and in some cases may exacerbate them (Eddy et al., 2011; Landi et al., 2018). Extensive evidence supports that some of the neuropathological mechanisms promoting memory loss in epilepsy include aberrant activation of glial, immune, and inflammatory pathways (Ravizza and Vezzani, 2018; Wyatt-Johnson and Brewster, 2020). However, the exact neuroimmune signaling mechanism underlying the cognitive dysfunction in epilepsy is still elusive. Our previous research identified the complement C3 as a potential candidate molecule (Schartz et al., 2018, 2019; Wyatt-Johnson and Brewster, 2020). In the current study, we investigated the role of complement C3 in memory loss induced by prolonged and continuous seizure activity, also known as status epilepticus (SE).

The complement system is part of the innate immune system, which acts as a protective barrier and first response mechanism against all antigens. In response to antigen stimulation, a well-orchestrated series of proteolytic reactions initiated by one of three pathways known as the classical, lectin, and alternative pathways results in the generation of biologically active complement proteins with a myriad of immune roles (Schartz and Tenner, 2020). These pathways converge to promote cleavage of C3, the central component of the complement system. Biologically active fragments, such as C3a and C3b/iC3b, play essential roles in regulating various processes, including inflammation (C3a), phagocytosis (C3b/iC3b), and the assembly of the membrane attack complex (MAC) (C3b) (Schartz and Tenner, 2020). In addition to these roles, C3 can guide synaptic pruning (Schafer et al., 2012). Notably, pathological conditions that promote increases in C3 (e.g., neurodegenerative disorders) are linked to exacerbated immune and inflammatory responses [reviewed in Schartz and Tenner (2020)], abnormal synaptic remodeling, and cognitive impairments (Hong et al., 2016; Vasek et al., 2016; Shi et al., 2017; Hammond et al., 2020; Werneburg et al., 2020; Zheng et al., 2022; Stokowska et al., 2023). While contemporary findings indicate that increases in C3 complement activation occur in seizure disorders [reviewed in Wyatt-Johnson and Brewster (2020)], the specific pathophysiological consequences in epilepsy remain to be elucidated.

Emerging evidence support that increases in immune complement signaling occur in human and pre-clinical models of epilepsy (reviewed in Brewster, 2019; Wyatt-Johnson and Brewster, 2020). High levels of complement molecules, including C3, are consistently found in brain biopsies derived from patients with different epilepsies with drug-resistant seizures such as focal cortical dysplasia (FCD) (Wyatt et al., 2017; Gruber et al., 2022), tuberous sclerosis complex (TSC) (Boer et al., 2008), and temporal lobe epilepsy (TLE) (Aronica et al., 2007). Evidence from experimental models suggest that seizures may contribute to pathological complement C3 deposition (Kharatishvili et al., 2014; Schartz et al., 2018, 2019; Wei et al., 2021). Increases in C3 protein levels appear following episodes of SE in rodent models of acquired TLE (Kharatishvili et al., 2014; Schartz et al., 2018, 2019). In these models, a single episode of SE promotes the development of unprovoked seizures along with learning and memory deficits that recapitulate the pathophysiology of human TLE (Levesque et al., 2015). We and others found that SE-induced increases in hippocampal C3 levels correlated with seizure severity and with the development of hippocampal-dependent memory deficits (Kharatishvili et al., 2014; Schartz et al., 2018, 2019). Based on these findings, we hypothesized that SE-induced increases in C3 activation may contribute to the development of memory impairments. To interrogate this idea, we used C3 knockout (KO) and wild type (WT) mice and assessed memory using novel object recognition (NOR) following SE induction with the chemoconvulsant pilocarpine.

## 2. Materials and methods

### 2.1. Animals

Homozygous pairing of wild type (WT) (JAX 0006640) or C3 KO (JAX 003641) mice (Jackson Labs) was used to produce experimental mice. Absence of C3 protein in the brains of C3 KO mice was validated using immunoblots. SE inductions were performed on male mice (2−3 months old) because we had previously established the specific spatiotemporal window for complement activation during the latent period of epileptogenesis in male rodents (Schartz et al., 2016, 2018, 2019). Our ongoing and future studies are focusing on determining the profile of complement activation in females and assessing sex as a biological variable in the context of SE and epilepsy. Mice were kept at ambient temperature with a 12-hr light-dark cycle (6:00−18:00) and unlimited access to food and water. Mice were group-housed (3−4/cage) at the Psychological Sciences animal facility at Purdue University or at the Laboratory Animal Research Core in the Dedman Life Sciences Building at Southern Methodist University. All procedures were approved by Purdue Animal Care and Use Committee (Protocol #1309000927) and Southern Methodist University IACUC (Protocol #A21-003-BREA**).** All animal research followed NIH and Institutional guidelines (Percie du Sert et al., 2020).

### 2.2. Pilocarpine-induced SE

Intraperitoneal (i.p.) injections of scopolamine methyl bromide (1 mg/kg; S8502-1G, Sigma-Aldrich, St. Louis, MO, United States) were given 30 min prior to pilocarpine (dissolved in sterile 0.9% saline) to induce SE (325−350 mg/kg, i.p. for dose response, 350 mg/kg for behavior cohorts) (P6503-10G, Sigma-Aldrich). Sham-controls were given saline. Diazepam (10 mg/kg, i.p; Hospira, Inc., Lake Forest, IL, United States) was injected approximately 1 h after SE onset to interrupt seizure activity. Mice were monitored continuously after pilocarpine administration and scored at 1 min intervals for the first 30 min, 2 min intervals between 30−40 min and 5 min intervals thereafter until Diazepam was administered. Seizures were scored according to the Racine scale (Racine, 1972). Modified Racine scale: (1 = rigid posture, mouth moving; 2 = tail clonus; 3 = partial body clonus, head bobbing; 4 = rearing; 4.5 = severe whole body clonic seizures while retaining posture; 5 = rearing and falling; 6 = tonic-clonic seizure with jumping or loss of posture). Mice were classified as SE when they reached a Racine score of ≥4.5. To aid in recovery, mice received 0.9% saline injections (i.p.), and supplementary chocolate Ensure and up to six Kellogg’s Fruit Loops pieces were put in the cages (control and SE mice) along with their rodent chow at 1 and 2 days after SE. Body weight was recorded daily for up to 2 weeks after SE. We injected 71 WT and 70 C3 KO mice with pilocarpine and 36 WT and 30 C3 KO mice with saline. Out of those injected with pilocarpine, 42.2% WT and 45.5% C3 KO mice died from SE-related complications. The final numbers of SE mice used in subsequent experiments were 41 WT and 38 C3 KO. The final numbers of control mice were 36 WT and 30 C3 KO.

### 2.3. Open field (OF)

Behavioral testing was performed between 2 and 3 weeks post SE. Prior to any behavioral testing, mice were handled for 1−2 min per day for 5 days to acclimate them to the experimenter. For the OF, mice were placed in an empty arena (40 × 40 × 30 cm) for 10 min. During this time all mice were recorded and tracked with Any-Maze V4.99 (Wood Dale, IL) to measure distance travelled, average speed, time freezing, and time spent in the inner and outer zones as indicators of general locomotor activity or anxiety-like behavioral differences between treatment groups.

### 2.4. Novel object recognition (NOR)

Novel object recognition (NOR) began 24 h after OF, where mice were allowed to explore two similar objects in the same chamber for 10 min (acquisition trial) (Trial 1), followed by a test trial in which one object was replaced by a novel object and mice were allowed to explore freely for 5 min (Trial 2). The inter-trial interval between acquisition and testing was set at 5 days, based on a pilot study of several intervals, and habituation and testing times (data not shown). Mice were returned to their home cages between trials. All trials took place under red lights. Object placement was counterbalanced to control for potential side bias. The arena and objects were cleaned with 70% ethanol between trials. Object exploration was assessed with Any-maze V4.99 and verified by blinded investigators. Discrimination index (DI) was calculated [(Novel-Familiar)/(Novel + Familiar) × 100]. Mice with DI ≥ ± 25 during acquisition were removed to eliminate mice with innate side preference (Schartz et al., 2018, 2019). This resulted in the exclusion of one WT + SE and one C3 KO + SE mouse from NOR analysis.

### 2.5. Western blot (WB)

Mice were euthanized at either 1 h or at 14 days after SE (cohorts separate from the behavior cohorts) with a lethal dose of Beuthenasia (200 mg/kg i.p.) and transcardially perfused with ice-cold phosphate buffered saline (PBS) for approximately 5 min or until the blood was cleared. Hippocampi were dissected and processed for immunoblotting as previously described (Schartz et al., 2018, 2019). Equal protein concentrations of whole lysates were resolved through SDS-PAGE, transferred to polyvinylidene difluoride membranes (88518, Thermo Scientific, Waltham, MA, United States), blocked with blocking solution [5% milk in 1X Tris buffered saline with 0.1% tween (0.1% 1X TBST)] (2 h RT) and incubated with primary antibodies overnight (4°C) or 2 h (RT). Primary antibodies used were rabbit anti-C3 (1:1K; ab200999, Abcam, Cambridge, United Kingdom), goat anti-C3 (1:100; 855730, MP Biomedical, Santa Ana, CA, United States) rabbit anti-phospho-S6 (1:1K; #5364, Cell Signaling), rabbit anti-S6 (1:1K; #2217, Cell Signaling, Danvers, MA, United States), mouse anti-PSD95 (1:10K; MABN68, Millipore, Burlington, MA, United States), mouse anti-GFAP (1:40K; #3670, Cell Signaling), rabbit anti-synaptophysin (1:1K; ab16659, Abcam), and rabbit anti-actin (1:5K; ab198991, Abcam) or anti-gapdh (1:30K; G9545, Sigma Aldrich) (loading control). After washing in 1X TBST, membranes were incubated in HRP-linked secondary anti-rabbit, mouse, or goat IgG antibodies for 1 h at RT. Membranes were stripped of primary antibodies using stripping buffer (25 mM glycine, pH 2.0, 10% SDS) for 1−2 h at RT, washed in 1X TBST and re-incubated with different primary antibodies. Immunoreactive signal was visualized with enhanced chemiluminescence (32106, Thermo Scientific) and captured on radiography film (BX57, MIDSCI, Fenton, MO, United States). Immunoreactive bands obstructed by bubbles or damaged due to gel breakage were not analyzed. Pixel intensity of the immunoreactive bands was measured using Image J software (NIH), subtracted from the background, normalized to the actin bands of the same sample/lane, and shown as percent change of controls.

### 2.6. Immunohistochemistry (IHC)

Mice were deeply anesthetized with Euthasol (200 mg/kg) and perfused with ice-cold 0.9% saline solution. Brains were post-fixed overnight in 4% paraformaldehyde, cryoprotected in 30% sucrose, frozen in dry ice, and stored at −80°C until used. Coronal brain sections (30 μm) were cut using a Leica CM1950 cryostat and stored in phosphate buffered saline (PBS) + 0.1% Sodium Azide at 4°C (PBS: BP399, Fisher Scientific). Colorimetric IHC was performed following previously described protocols (Schartz et al., 2016, 2018). Briefly, sections were incubated in 1XPBS (5 min), 3% hydrogen peroxide (30 min), 1X PBS with 0.3% Triton (1X PBS-0.3T) (20 min), and in immuno buffer (5% goat serum, 0.3% BSA, 0.3% 1X PBS-0.1T) (1 h) at RT. Incubation with the primary antibody anti-mouse GFAP (1:1000; #3670, Cell Signaling) was done overnight on a rotating platform at 4°C. Following washes in 0.1% 1X PBST (3 × 10 min), sections were incubated in biotinylated goat anti-mouse secondary antibody (1:500; BA-9200, Vector Laboratories) (1 h), washed with 0.1% 1X PBST (3 × 10 min), and incubated with ABC Peroxidase Standard Staining Kit (#32020, ThermoFisher) (30 min). Immunoreactive GFAP signal was visualized using the DAB peroxidase (HRP) Substrate Kit, 3,3’-diaminobenzidine according to manufacturer’s instructions (SK-4100, Vector Laboratories). Sections were mounted on gelatin-coated slides, air dried, dehydrated through increasing alcohol concentrations (50, 70, 95, and 100%), de-fatted in Xylene (2 × 3 min), and cover slipped using Permount mounting media. Images (4-6 sections per brain) were captured using a Nikon Ti2-E Inverted Motorized Microscope.

### 2.7. Statistical analyses

Sample sizes were determined with G*Power using previously gathered data to calculate the number of mice per group needed to achieve power of 0.8. NOR object preference was analyzed using paired *t*-tests. Data acquired from OF, DI, and NOR were compared using two-way ANOVA with Tukey’s *post hoc* test using GraphPad Prism Software version 8.0.1. All immunoblot data were compared using independent *t*-tests (2 groups) or one-way ANOVA with Sidak’s multiple comparison test (>2 groups). Significance was set at *p* = 0.05.

## 3. Results

### 3.1. C3 KO mice develop pilocarpine-induced SE

**FIGURE 1:**
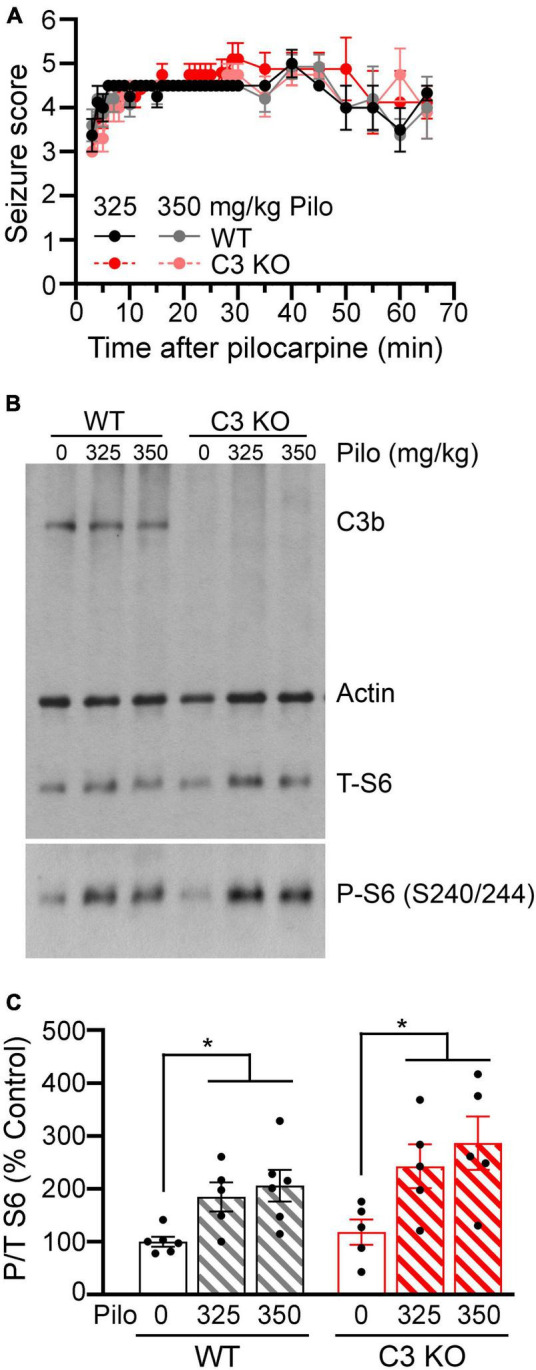
Wild type (WT) and C3 KO mice show similar behavioral seizure responses to pilocarpine. **(A)** Pilocarpine given at 325 or 350 mg/kg to WT and C3 knockout (KO) mice induced behavioral seizures according to a modified Racine scale (1 = rigid posture, mouth moving; 2 = tail clonus; 3 = partial body clonus, head bobbing; 4 = rearing; 4.5 = severe whole body clonic seizures while retaining posture; 5 = rearing and falling; 6 = tonic-clonic seizure with jumping or loss of posture). Pilo 325 mg/kg: WT, *n* = 5; C3 KO, *n* = 6. Pilo 350 mg/kg: WT, *n* = 7; C3 KO, *n* = 6. **(B)** Immunoblotting was used to determine hippocampal protein levels of complement C3b, phosphorylated (P) and total (T) ribosomal S6 protein, and actin (loading control) at 1 h after pilocarpine-induced status epilepticus (SE) in WT and C3 KO mice. **(C)** Quantification of the immunoreactive bands is shown as percent control for P/T S6 ratio as a measure of pilocarpine-induced neuronal activity in whole hippocampal homogenates (*n* = 5–6/group). Data were compared using two-way ANOVA with Sidak’s multiple comparisons test. Data are shown as mean ± SEM. *p < 0.05.

The first aim of this study was to confirm induction of SE by pilocarpine in C3 KO and WT mice. We performed SE inductions on 2−3-month-old mice. At this age, C3 KO and WT mice have been shown to have similar spine density and synaptic responses in the hippocampus (Perez-Alcazar et al., 2014; Shi et al., 2015). Therefore, we performed a dose response with either 325 or 350 mg/kg of pilocarpine (Figures 1A, B). Both C3 KO and WT mice reached similar seizure scores according to the Racine scale (Racine, 1972) with 325 mg/kg [*F* (1, 9) = 0.06, *p* = 0.81] or 350 mg/kg [*F* (1, 11) = 1.84, *p* = 0.20] of pilocarpine (Figure 1A), suggesting that behavioral seizure susceptibility to pilocarpine was not altered by the lack of C3 protein.

To confirm that the initial neural responses to pilocarpine-induced SE were similar between WT and C3 KO, we collected hippocampi from a subset of animals at 1 h during SE. First, we confirmed the lack of C3 protein in the C3 KO hippocampal lysates relative to the WT group using antibodies against C3 (Figure 1B). Next, we determined levels of phosphorylated S6 (P-S6) relative to total S6 (P-S6/T-S6), a protein within the mechanistic target of rapamycin complex 1 pathway that is indicative of increased neuronal activity by chemoconvulsants (Zeng et al., 2009; Brewster et al., 2013; Figure 1C). Similar SE-induced increases in the P-S6/T-S6 ratio were evident in hippocampi of WT mice [*F* (2, 14) = 5.75, *p* = 0.02] and C3 KO mice [*F* (2, 12) = 4.71, *p* = 0.03], suggesting hippocampal neuronal activation may be similar in both WT and C3 KO animals subjected to pilocarpine-induced SE. *Post-hoc* analysis showed that the 350 mg/kg dose significantly increased P-S6/TS6 in WT (*p* = 0.01) and C3 KO (*p* = 0.02) mice. Thus, hereafter a dose of 350 mg/kg of pilocarpine was used to induce SE in animals used for subsequent behavioral assessments.

**FIGURE 2:**
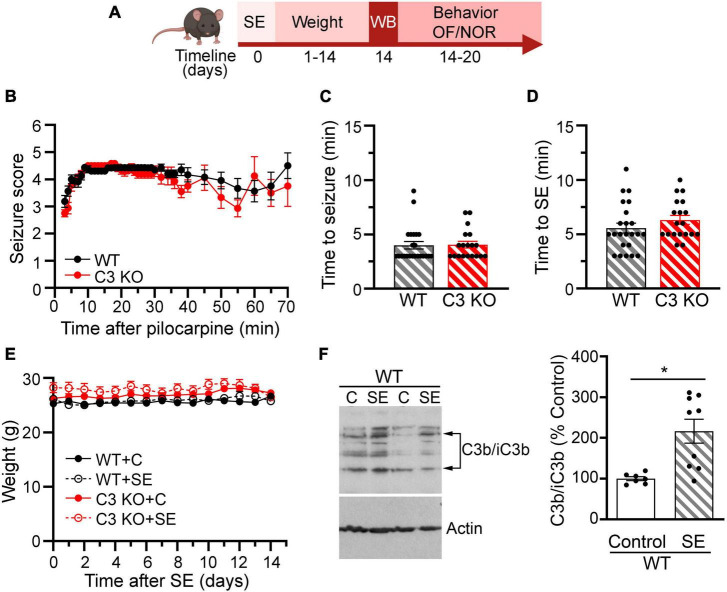
Effects of pilocarpine-induced SE were assessed through weight, behavior, and immunoblots in wild type (WT) or C3 knockout (KO) mice. **(A)** Diagram with the experimental design showing the timeline of SE induction (day 0), assessment of C3 activation with western blot (WB) (day 14), daily weight measurements (days 1–14), and behavioral tests for open field (OF) and novel object recognition (NOR). Created with BioRender.com. **(B)**, Behavioral seizures were monitored for 65–70 min after SE induction and scored according to the Racine scale (RS) (1: rigid posture, mouth moving; 2: tail clonus; 3: partial body clonus, head bobbing; 4: rearing; 4.5: severe whole body clonic seizures while retaining posture; 5: rearing and falling; 6: tonic-clonic seizure with jumping or loss of posture). **(C)** Time to first seizure (level 3). **(D)** Time to SE (level 4.5). WT, *n* = 23; C3 KO, *n* = 19. **(E)** Daily body weight for mice in each group WT + C, WT + SE, C3 KO + C, and C3 KO + SE are shown. WT + C, *n* = 14; WT + SE, *n* = 14; C3 KO + C, *n* = 14; C3 KO + SE, *n* = 14. Data were compared using two-way ANOVA with Tukey’s *post hoc* test. **(F)** Representative immunoblots and quantification of the protein levels of the fragments of C3b/iC3b and actin (loading control) at 2 weeks following SE inductions in whole hippocampal homogenates derived from WT mice (WT + C, *n* = 7; WT + SE, *n* = 9). Data were compared using independent *t*-tests. Data are shown as mean ± SEM. *p < 0.05.

**FIGURE 3:**
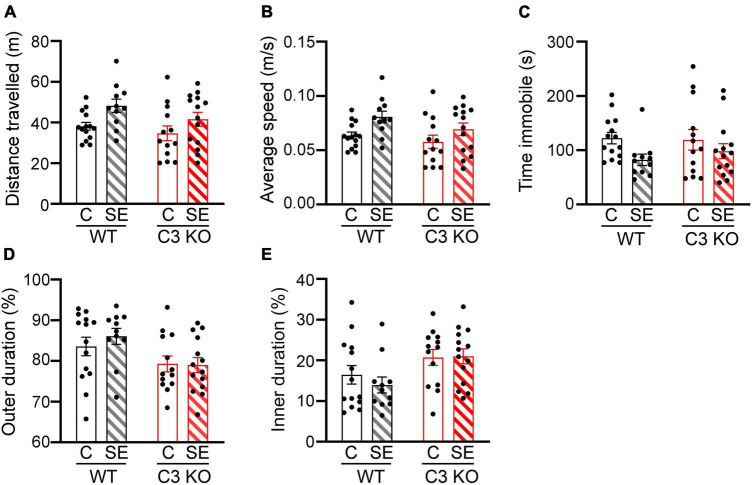
SE does not significantly alter locomotion or anxiety-like behaviors. The open field (OF) test was used to assess locomotion and anxiety-like behaviors between wild type (WT) and C3 Knockout (KO) mice subjected to status epilepticus (SE) or control (C) sham conditions (WT + C, WT + SE, C3 KO + C, C3 KO + SE). **(A–D)**, Measurements of distance travelled (meters, m) **(A)**, average speed (m/s) **(B)**, time spent immobile (seconds, s) **(C)**, and percentage of time spent in the outer **(D),** and inner **(E)** zones of the arena are shown. Data were compared using two-way ANOVA with Tukey’s *post hoc* test. WT + C, *n* = 14; WT + SE, *n* = 11; C3 KO + C, *n* = 14; C3 KO + SE, *n* = 14. Data shown as mean ± SEM.

**FIGURE 4:**
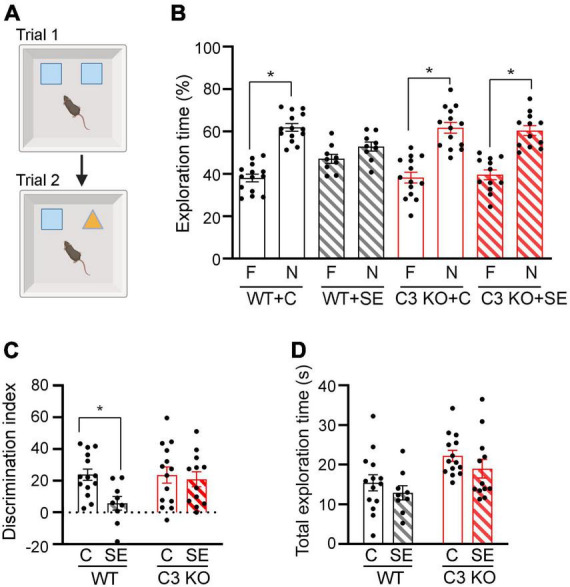
C3 knockout (KO) mice are protected against SE-induced recognition memory deficits. **(A)** Diagram of the novel object recognition test used to compare memory between wild type (WT) and C3 KO mice subjected to status epilepticus (SE) or control (C) sham conditions (WT + C, WT + SE, C3 KO + C, C3 KO + SE). Created with BioRender.com. **(B)** Percent exploration time of the familiar (F) object vs. novel (N) object compared within treatment groups (paired *t*-test) is shown for trial 2. **(C)** Discrimination index (DI) [(novel-familiar)/(novel + familiar) × 100] compared discrimination of the novel object between treatment groups (two-way ANOVA). **(D)** Total exploration of objects compared exploratory behaviors between treatment groups by two-way ANOVA with Tukey’s *post hoc* test. WT + C, *n* = 14; WT + SE, *n* = 9; C3 KO + C, *n* = 14; C3 KO + SE, *n* = 12. Data are shown as mean ± SEM. *p < 0.05.

To monitor the health and recovery of all WT and C3 KO mice after SE or sham treatment, mice were weighed daily for 2 weeks (Figure 2A). To assess behavioral comorbidities, we evaluated anxiety-like behaviors (Figure 3), and memory (Figure 4) between 2 and 3 weeks after SE. We found that WT and C3 KO mice used for behavioral evaluations developed SE at similar Racine levels following pilocarpine injections (350 mg/kg) (Figure 2B). The time to first seizure (Racine scale 3) (Figure 2C) and SE (Racine scale ≥ 4.5) (Figure 2D) were similar between the WT and C3 KO groups. Mice from all groups had consistent body weight throughout the study (Figure 2E). Two weeks after SE, we determined the protein levels of C3 in hippocampi from a subset of WT mice with or without SE (Figure 2F). Activated C3 is cleaved into smaller fragments that include C3b/iC3b (Schartz et al., 2018). Utilizing an antibody that recognizes all cleavage products of C3, we found that the levels of C3b/iC3b protein were significantly increased in the SE group compared to controls (*p* = 0.02) (Figure 2F), consistent with our previous findings in rats (Schartz et al., 2018, 2019) and human (Wyatt et al., 2017). Taken together these findings support that SE provokes increases in C3 activation.

### 3.2. C3 KO mice do not develop SE-induced recognition memory deficits

To assess potential differences in locomotion and anxiety-like behaviors in the different groups of mice, we compared total distance travelled (Figure 3A), average speed (Figure 3B), time spent immobile (time freezing) (Figure 3C), and time spent around the outer walls zone (Figure 3D) or in the inner zone of the arena (Figure 3E) between WT + C, C3 KO + C, WT + SE and C3 KO + SE mice. WT + C and WT + SE mice showed no significant differences in these measures although WT + SE mice showed a slight but not significant increase in distance travelled (*p* = 0.09), faster speed (*p* = 0.08), and less time freezing (*p* = 0.06) compared to WT + C mice. The locomotion and anxiety-like behaviors were not different between C3 KO + C and C3 KO + SE mice (*p* > 0.05). There was no effect of genotype or treatment on time spent in the inner or outer zones of the arena. These findings indicate that at 2 weeks after SE, hyperactivity and anxiety-like behaviors were not significantly altered in either WT or C3 KO mice.

We and others previously reported that a single episode of SE results in memory deficits that are evident 2 weeks post-SE induction (Brewster et al., 2013; Schipper et al., 2016; Schartz et al., 2018). Therefore, at this time point we determined recognition memory using NOR (Figure 4). During NOR acquisition, all groups showed similar exploration times of the right or left objects (data not shown), and the DI or total exploration time were not different between the groups (Two-way ANOVA, *p* > 0.05) (data not shown). During the test trial (Trial 2), WT + C mice spent significantly more time exploring the novel object compared to the familiar one [*t* (13) = 6.64, *p* < 0.001], as did C3 KO + C mice [*t* (13) = 4.60, *p* < 0.001] (Figure 4B). WT + SE mice did not show exploratory preference for either object [*t* (8) = 1.34, *p* = 0.22], suggesting a recognition memory deficit in this mouse group. In contrast, mice from the C3 KO + SE group explored the novel object more than the familiar object [*t* (11) = 4.45, *p* = 0.001] (Figure 4B). In addition, we found a significant effect of treatment on DI [*F* (1, 45) = 5.02, *p* = 0.03] (Figure 4C), but no effect of genotype [*F* (1, 45) = 2.55, *p* = 0.12] and no interaction between treatment and genotype [*F* (1, 45) = 2.79, *p* = 0.10]. Interestingly, our data indicate that C3 KO mice spent more time exploring both objects overall [*F* (1, 45) = 10.76, *p* = 0.002] (Figure 4D), which may contribute to increased learning.

### 3.3. C3 KO mice are protected against SE-induced increases in GFAP

**FIGURE 5:**
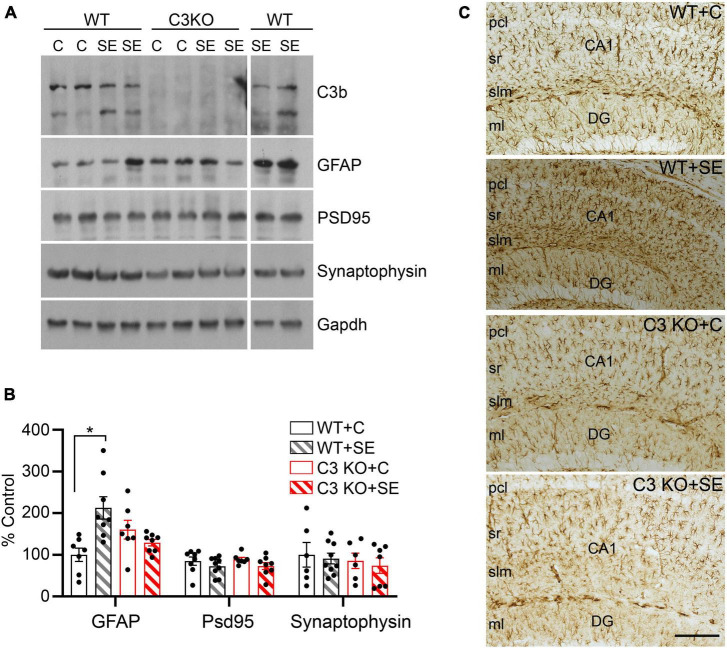
C3 knockout (KO) mice are protected against SE-induced increases in hippocampal GFAP protein levels. **(A)** Hippocampal whole lysates derived from WT + C, WT + SE, C3 KO + C, and C3 KO + SE mice (2 weeks post-SE) were processed for western blotting and probed with antibodies against C3, GFAP, PSD95, synaptophysin, and corresponding Gapdh (loading control). **(B)** Quantitative analysis shown as percent control (WT + C) for GFAP, PSD95, and synaptophysin. WT + C, *n* = 6–8; WT + SE, *n* = 8–9; C3 KO + C, *n* = 6–7; C3 KO + SE, *n* = 7–8. Data shown as mean ± SEM. *p < 0.05 by one-way ANOVA with Sidak’s multiple comparison test. The uncropped immunoblots are shown in the Supplementary Figure 1. **(C)** Representative images with GFAP immunostaining in hippocampi from WT + C, WT + SE, C3 KO + C, and C3 KO + SE mice (2 weeks post-SE) are shown (*n* = 4/group). pcl, pyramidal cell layer; sr, stratum radiatum; slm, stratum lacunosum-moleculare; DG ml, dentate gyrus molecular layer. Scale bar, 200 μm.

Given that the main sources of C3 in the brain are astrocytes (Lian et al., 2015; Liddelow et al., 2017) and that C3 can contribute to the modulation of synaptic elements (Stevens et al., 2007; Schafer et al., 2012), we measured the relative protein levels of the pan-reactive astrocytic marker GFAP along with synaptic markers PSD95 and synaptophysin in hippocampal homogenates at 14 days after SE (Figure 5). We found significant increases in the protein levels of GFAP in the WT + SE group compared to WT + C (*p* = 0.037) but not in C3 KO + SE compared to C3 KO + C or WT + C groups (*p* > 0.05) (Figures 5A, B). The levels of PSD95 or synaptophysin were not different between the groups. Consistent with these GFAP findings in whole hippocampal lysates, immunohistological analysis showed a denser population of GFAP-positive cells in hippocampi of WT + SE mice compared to WT + C, C3 KO + C, and C3 KO + SE groups (Figure 5C). Taken together these data support that C3 KO mice are protected against SE-induced increases in GFAP suggesting that C3 modulation of astrocyte function may play a role in the associated memory defects (Figure 4).

## 4. Discussion

Currently available anti-seizure drugs do not address, and can often exacerbate, intellectual disabilities (Thijs et al., 2019), highlighting a need for novel therapeutics targets to improve cognitive function in epilepsy. Our goal is to identify molecular targets that can be modulated for the treatment and control of cognitive comorbidities in epilepsy. Previously, we found that SE-induced C3 activation paralleled memory decline after SE in rats (Schartz et al., 2018, 2019). Here we confirm the association between SE-induced increased C3 levels (Figure 2) and memory deficits (Figure 4) in WT mice. We found that C3 KO mice subjected to SE did not develop recognition memory deficits (Figure 4) and that locomotor or anxiety-like behaviors were not different between the groups (Figure 3). Further, the lack of C3 protected against SE-induced increases in hippocampal GFAP protein levels (Figure 5) suggesting that C3 may modulate astrocyte responses to prolonged seizures.

C3 signaling is associated with the modulation of cognitive performance under both physiological and pathological conditions (Schartz and Tenner, 2020). Under normal conditions, young C3 KO mice (2−3-month-old) showed enhanced hippocampal-dependent spatial learning relative to age-matched WT mice (Perez-Alcazar et al., 2014), and aged C3 KO mice (at 16 months) demonstrated better spatial and contextual memory relative to age-matched WT mice (Shi et al., 2015). It is also well established that activation of the immune complement system can occur as a result of brain injury and in neurodegenerative disorders (Schartz and Tenner, 2020). Under pathological conditions such as those associated with traumatic brain injury, multiple sclerosis, and Alzheimer’s disease (AD), complement proteins, including C3, are linked to impaired cognitive functions (Carpanini et al., 2019; Schartz and Tenner, 2020; Bourel et al., 2021; Saez-Calveras et al., 2022). In this study, we found SE-induced impairments in recognition memory in WT mice but not in C3 KO mice (Figure 4). This implies that increased C3 signaling disrupts neural processes that regulate cognitive function in the context of SE. Our finding is further supported by studies in mouse models of controlled cortical impact, where pharmacological suppression of C3 or genetic ablation of C4 protected against the development of hippocampal-dependent spatial learning and memory defects (You et al., 2007; Mallah et al., 2021). Similarly, drug-mediated inhibition or ablation of C3 signaling prevented or attenuated memory decline in experimental models of autoimmune encephalomyelitis (Hammond et al., 2020), West Nile virus infection (Vasek et al., 2016), and AD (Hong et al., 2016; Shi et al., 2017). To our knowledge, this study is the first to show that C3 KO mice are protected against recognition memory impairments in the context of SE.

Upon activation of the complement system, C3 is cleaved into C3a and C3b fragments. C3a regulates recruitment of leukocytes and microglia to the site of injury (Coulthard and Woodruff, 2015; Wu et al., 2016; Propson et al., 2021), and has also been shown to increase astrogliosis and exacerbate pathogenesis in a model of AD (Lian et al., 2016). C3b acts as an opsonin that promotes elimination of neuronal blebs/debris and guides microglial synaptic pruning (Schafer et al., 2012). However, C3b also contributes to the formation of C5 convertase, which cleaves the C5 protein into the potent anaphylatoxin C5a, and C5b which acts as the initiating molecule of MAC. In addition, complement C3 is characteristic of neurotoxic “A1” astrocytes (Liddelow et al., 2017) and its production in astrocytes is upregulated in aging and in disease (Boisvert et al., 2018; Propson et al., 2021) and in experimental epilepsy (Wei et al., 2021; Vasanthi et al., 2023). Thus, activation of C3 signaling can have multiple effects that are dependent on which cell type and downstream pathway it triggers resulting in modulation inflammatory and/or phagocytic responses.

In the context of inflammation, the communication between astrocytes and microglia via C3a/C3aR can trigger either pro-inflammatory or anti-inflammatory actions. In cases of acute peripheral inflammation, C3a activity promotes the infiltration of peripheral lymphocytes into the brain (Propson et al., 2021). C3a also promotes CD8 + T cell infiltration and microglial reactivity in aging mice (Propson et al., 2021). Ablation of C3aR1 prevents disruption of the BBB and attenuates the microglial response in these models. In the acute stage of ischemic stroke, increased levels of C3 signaling through C3aR1 are associated with worsening pathology and can lead to the development of epilepsy (Pekna et al., 2021). In the kainic acid model of SE, treatment with captopril, an angiotensin-converting enzyme inhibitor, reduced SE-induced activation of C3 signaling along with microgliosis, astrogliosis, and decreased the levels of inflammatory cytokines including IL-1β, IL-18, and TNF-α in the hippocampus (Dong et al., 2022). In a rat soman model of SE and acquired epilepsy, attenuation of C3 expression in reactive astrocytes as well as reductions in microgliosis and inflammation were coupled to improved cognitive function following a treatment with 1400W, a highly selective inhibitor of inducible nitric oxide synthase (Vasanthi et al., 2023). These effects may be due to the broad overlap between microglia and astrocytes in the hippocampus, which may be mediated, in part, by C3/C3aR signaling in the context of SE (Wei et al., 2021). Thus, our finding that SE-induced increases in hippocampal GFAP protein levels did not occur in C3 KO mice (Figure 5) suggest that augmented C3 signaling may potentially be implicated in modifying astrocytic functions. This possibility extends to a potential involvement of C3 in the interplay between microglia and astrocytes, as well as in the regulation of inflammation after SE and in chronic epilepsy (Wei et al., 2021; Vasanthi et al., 2023), which we will interrogate in follow up studies.

In addition to inflammatory control, C3 has been implicated in microglia-mediated synaptic pruning under physiological and pathological conditions (Carpanini et al., 2019; Prinz et al., 2019; Wang et al., 2020). In the healthy brain, C3 contributes to the refinement of synaptic circuits within the visual system (Stevens et al., 2007; Schafer et al., 2012) and the formation of astrocytic and vascular networks (Gnanaguru et al., 2023). However, in some neurodegenerative disorders characterized by cognitive decline, high levels of C3 are associated with exacerbated synaptic loss (Hong et al., 2016; Vasek et al., 2016; Shi et al., 2017; Alawieh et al., 2018; Krukowski et al., 2018; Prinz et al., 2019; Hammond et al., 2020; Werneburg et al., 2020). In a model of acquired TLE, C3 depletion after SE reduced microglial field area and attenuated neuronal loss (Wei et al., 2021). While it is possible that the prevention of SE-induced memory deficits in C3 KO mice may interfere with synaptic remodeling, here we did not find alterations in the levels of PSD95 or synaptophysin in hippocampi of WT or C3 KO mice subjected to SE.

It is important to note that the consequences of C3 activation may be highly dependent on context, time, and sex. We and others found that increased complement activation, including C3 activation correlated with increased seizure frequency (Xiong et al., 2003; Kharatishvili et al., 2014; Schartz et al., 2018; Wyatt-Johnson and Brewster, 2020). While we did not observe differences in latency to behavioral seizures or SE between WT and C3 KO mice, it is possible that subtle differences in electrographic seizure activity could contribute to epileptogenesis. Potential differences in electrographic activity could be attributed to inherent genetic distinctions associated with the use of WT and C3 KO mice that were not from the same litter. However, it is noteworthy that homozygous C3 KO and WT mice exhibited comparable AMPAR-mediated EPSC responses, and naïve C3 KO mice did not exhibit any spontaneous epileptiform activity in the hippocampus (Perez-Alcazar et al., 2014). Furthermore, evidence suggests that blocking downstream complement components such as C3 and C5 (and their receptors) reduces seizure severity and susceptibility (Libbey et al., 2010; Benson et al., 2015). Thus, it may be possible that C3 KO mice are protected against SE-induced epileptogenesis and subsequent generation of unprovoked seizures. This protective effect, in turn, might contribute to the prevention of recognition memory deficits and the reduction of hippocampal GFAP levels. Another limitation of this study is that we did not assess sex as a biological variable. Although human studies indicate that the risk of SE is similar between males and females (Baumann et al., 2023), recent findings suggest that SE is associated with long-lasting sex-dependent differences in brain structure and function (Gage et al., 2023). Additionally, there is emerging evidence indicating that immune signaling pathways can vary based on sex (Osborne et al., 2018). Therefore, it is possible that the extent of complement activation, neuroimmune dysfunction and pathophysiological sequalae of SE may be sex dependent and requires further investigation.

In summary, complement C3 is a reliable marker of brain injury and neurodegenerative diseases (Schartz and Tenner, 2020). Here, we show evidence supporting that C3 KO mice were protected against SE-induced recognition memory impairments and that dysregulation of astrocytes may play a role. Taken together, our findings suggest that C3 is likely a disease-enhancing molecule in the context of SE-induced brain injury.

## Data availability statement

The original contributions presented in this study are included in the article/Supplementary material, further inquiries can be directed to the corresponding author.

## Ethics statement

The animal study was approved by the Purdue University IACUC and the Southern Methodist University IACUC. The study was conducted in accordance with the local legislation and institutional requirements.

## Author contributions

NS: Conceptualization, Data curation, Formal analysis, Writing – original draft, Writing – review and editing, Investigation, Methodology, Validation. AA: Investigation, Methodology, Writing – review and editing. YL: Investigation, Methodology, Writing – review and editing. NP-H: Investigation, Methodology, Writing – review and editing. AB: Writing – review and editing, Conceptualization, Data curation, Formal analysis, Funding acquisition, Resources, Supervision, Writing – original draft.
