# Supplementary figures and images for: Mice deficient in complement C3 are protected against recognition memory deficits and astrogliosis induced by status epilepticus
Source: Front Mol Neurosci. 2023 Nov 14;16:1265944. doi: 10.3389/fnmol.2023.1265944 (PMC10682718; doi:10.3389/fnmol.2023.1265944)

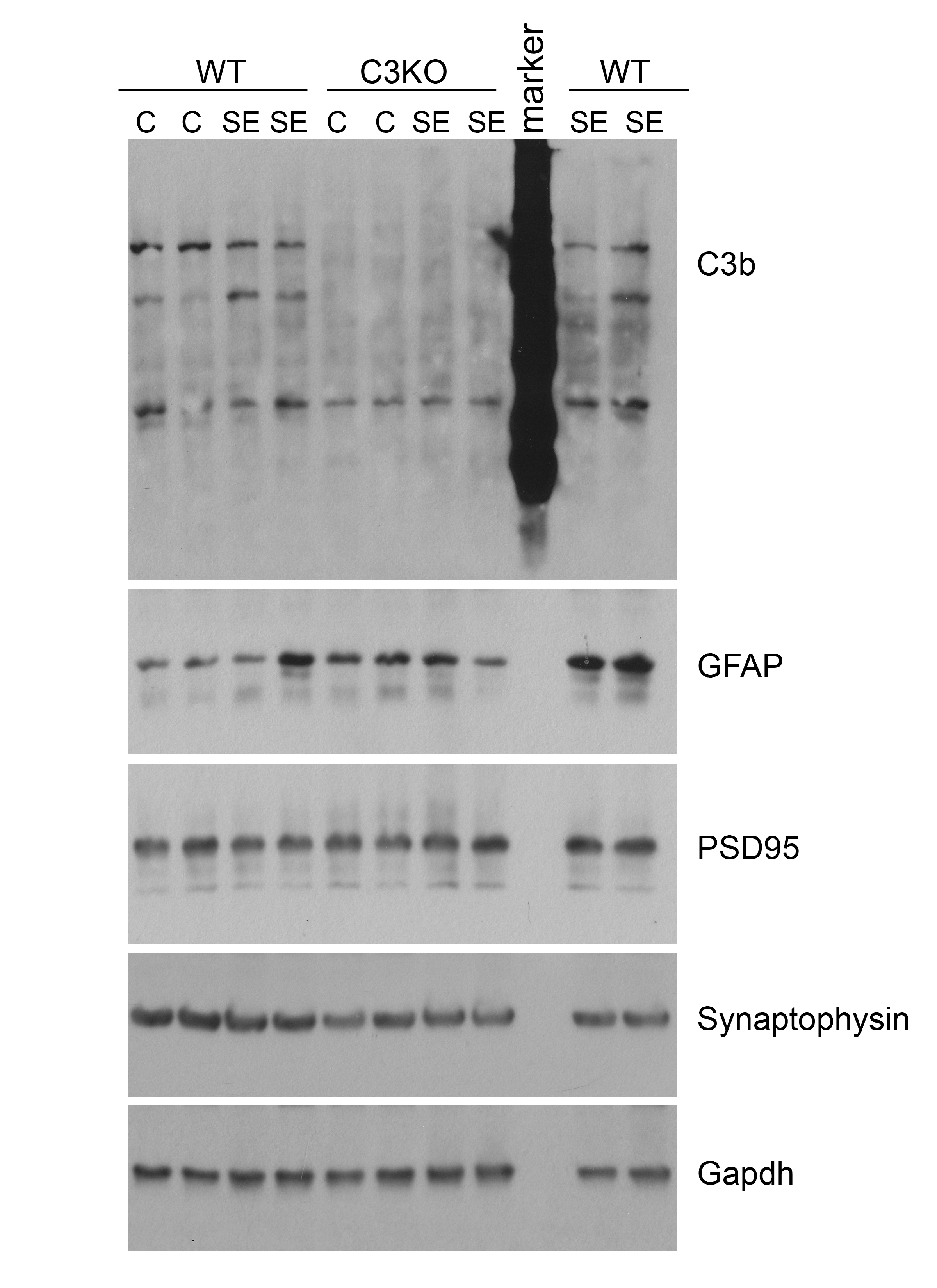

Supplement: Supplementary file 1 [file Image_1.JPEG]
